# Severe tooth wear in Prader-Willi syndrome. A case–control study

**DOI:** 10.1186/1472-6831-12-12

**Published:** 2012-05-28

**Authors:** Ronnaug Saeves, Ivar Espelid, Kari Storhaug, Leiv Sandvik, Hilde Nordgarden

**Affiliations:** 1 TAKO-centre, Lovisenberg Diakonale Hospital, Lovisenberggt 17, 0440 Oslo, Norway; 2 Faculty of Dentistry, University of Oslo, Oslo, Norway

**Keywords:** Prader-Willi syndrome, Tooth wear, Tooth grinding, Saliva secretion, Rehabilitation

## Abstract

**Background:**

Prader-Willi syndrome (PWS) is a rare complex multsystemic genetic disorder characterized by severe neonatal hypotonia, endocrine disturbances, hyperphagia and obesity, mild mental retardation, learning disabilities, facial dysmorphology and oral abnormalities. The purpose of the present study was to explore the prevalence of tooth wear and possible risk factors in individuals with Prader-Willi syndrome.

**Methods:**

Forty-nine individuals (6-40 years) with PWS and an age- and sex-matched control group were included. Tooth wear was evaluated from dental casts and intraoral photographs and rated by four examiners using the Visual Erosion Dental Examination (VEDE) scoring system and the individual tooth wear index I_A_. In accordance with the VEDE scoring system, tooth wear was also evaluated clinically. Whole saliva was collected.

**Results:**

Mean VEDE score was 1.70 ± 1.44 in the PWS group and 0.46 ± 0.36 in the control group (p < 0.001). Median I_A_ was 7.50 (2.60-30.70) in the PWS group and 2.60 (0.90-4.70) among controls (p < 0.001). In the PWS group tooth wear correlated significantly with age (VEDE; r = 0.79, p < 0.001, I_A_; r = 0.82, p < 0.001) and saliva secretion (VEDE; r = 0.46, p = 0.001, I_A_; r = 0.43, p = 0.002). Tooth grinding was also associated with tooth wear in the PWS group, as indicated by the mean VEDE 2.67 ± 1.62 in grinders and 1.14 ± 0.97 in non-grinders (p = 0.001) and median I_A_ values 25.70 (5.48-68.55) in grinders and 5.70 (1.60-9.10) in non-grinders (p = 0.003). Multivariate linear regression analysis was performed with tooth wear as the dependent variable and PWS (yes/no), age, tooth grinding and saliva secretion as independent variables. PWS (yes/no), age and tooth grinding retained a significant association with tooth wear, VEDE (p < 0.001) and log I_A_ (p < 0.001). The only factor significantly associated with tooth wear in the control group was age.

**Conclusions:**

Our study provides evidence that tooth wear, in terms of both erosion and attrition, is a severe problem in Prader-Willi syndrome. There is therefore considerable need for prosthodontic rehabilitation in young adults with PWS.

## Background

Prader-Willi syndrome (PWS) is the most common genetic human obesity syndrome. Recent epidemiological surveys estimate birth incidence to be at least 1:30000 and population prevalence up to 1:52000 [1-4]. The syndrome affects both males and females equally [1,5-7]. PWS is a complex multi-system disorder arising from lack of expression of paternally inherited genes on chromosome 15q11-q13 [7-11]. PWS develops if the paternal alleles are defective, missing or silenced. There is paternal deletion of chromosome 15q11-q13 in 70% of cases, maternal uniparental disomy (UPD) in 25%, and imprinting defects in 1% of cases [7,12,13].

The syndrome has a characteristic phenotype [14,15] which includes neonatal and infantile hypotonia, early feeding problems (naso-gastric tube feeding for more than two months), childhood onset hyperphagia, obesity, short stature associated with growth hormone (GH) deficiency, high pain threshold, learning disabilities and behavioral problems. Excessive daytime sleepiness and sleep apnea are common in individuals with PWS [16-18]. A narrow forehead, almond-shaped eyes, down-turned corners of the mouth and a thin upper lip are characteristic facial features. Varying degrees of oral motor dysfunction is common in individuals with PWS [19].

Thick, viscous saliva has been reported to be a consistent finding in PWS [20-22] and a diagnostic indicator of PWS in neonates [23]. Decreased salivary flow rates [24] and increased amounts of salivary ions and proteins have also been reported [20,21]. Dental caries [25-28], enamel defects [27-31] and poor oral hygiene [27,28] have been described in case reports. However, compared with previous reports, recent studies have identified more favourable oral health with respect to caries experience [22,24].

Progressive dental tissue loss (tooth wear) has been reported in PWS [22,32] and gastro-oesophageal reflux described in one case report [27]. To our knowledge, no studies on tooth wear in a PWS population compared with a control group have been published.

The present study is part of a comprehensive survey. The aim of this study was to explore the prevalence of tooth wear and associated risk factors in individuals with Prader-Willi syndrome. The null hypothesis predicted no difference in prevalence of tooth wear between individuals in the PWS group and an age- and sex matched control group.

## Methods

The study was carried out at the TAKO-centre, Lovisenberg Diakonale Hospital and at the Faculty of Dentistry, University of Oslo. The TAKO-centre is a national resource centre for oral health in rare medical conditions (frequency of less than 1:10 000). This study used an observational, matched case–control design.

### Ethical approval

The study protocol was approved by the Regional Committee for Medical Research Ethics and The Norwegian Data Inspectorate. Informed consent was obtained from all participants. When participants were under 18 years of age or were adults with a guardian, informed consent was also obtained from the parents or guardian.

### Study participants

Participants were recruited through the Norwegian Prader-Willi syndrome association. All association members aged 5 years and older (n = 95) received written information, designed for both children and adults, describing the study. Fifty-four individuals with PWS responded. Two of those who initially agreed to participate, later changed their minds due to the long travelling distance to the clinic. Two individuals were excluded after undergoing a new genetic test (negative Multiplex Ligation-dependent Probe Amplification), whilst one participant had only primary teeth and was therefore excluded from this part of the study. The final study group comprised 49 individuals (24 F, 25 M), age range 6.5-40.9 years. The examinations took place between January 2007 and April 2009. Confirmation of the genetic diagnosis of PWS was obtained from 3 different medical genetic centres. The participants came from all over Norway, although five children (6-13 years) in the study group were not ethnic Norwegians, as their parents came from Africa, Asia and Eastern Europe.

The control group was age- and sex-matched to the PWS group. Twenty-three healthy, unmedicated control participants (age 6-18 years) were recruited among patients at the Department of Pediatric Dentistry, Faculty of Dentistry, University of Oslo and 26 healthy adults were recruited among the staff at the Lovisenberg Diakonale Hospital. None of the control persons were dental personnel, and they were not expected to have more knowledge of dental health than the general population. All individuals with PWS and 26 of the controls were examined at the TAKO-centre, while the remaining 23 controls (6-18 years) were examined at the Department of Pediatric Dentistry, University of Oslo.

### Questionnaires

All participants with PWS underwent a thorough, structured anamnestic interview either during the consultation or, if parents did not attend the consultation, by telephone. The interview focused on oral and general health, sleep disorders, gastric reflux, medications (e.g. use of growth hormone) and nutrition. The frequency of consumption of acidic food and drinks was categorized as follows: More than once daily; once daily; several times per week; once per week; or never. Information about tooth grinding was also obtained. A modified questionnaire, omitting questions not relevant for a healthy control group, was given to the control group prior to the examination. Dental records, including radiographs, from all study participants and controls under 19 years of age, were recovered from the public dental health service where all individuals under the age of 19 years and all with mental retardation regardless of age receive free dental care.

### Clinical assessments

Prior to the examination, all participants (and/or their carers) received written information and photographs describing the procedures. Study participants and controls were examined once by the same examiner (RS). Intraoral photographs were taken of all participants and dental impressions (alginate) were taken of 48 participants from each group. Dental impressions were not taken of two individuals due to cooperation problems (PWS, n = 1) or ongoing orthodontic treatment with fixed appliances (control, n = 1).

Tooth wear was evaluated using two indices, the Visual Erosion Dental Examination (VEDE) scoring system [33] and a modified individual tooth wear index (I_A_) [34]. In the study group tooth wear was evaluated clinically by RS according to the VEDE scoring system. The VEDE-index, a modification of the dental erosion index proposed by Lussi [35], is a 6-point scoring system which contains a visual guide with clinical photos: 0 = no erosive wear; 1 = loss of enamel surface characteristics; 2 = loss of enamel surface contour; 3 = loss of dentine from less than one-third of the surface; 4 = loss of dentine from more than one-third and less than two-thirds of the surface; 5 = loss of dentine from more than two-thirds of the surface. Tooth wear was evaluated for each permanent tooth surface (buccal/labial, occlusal/incisal and palatinal/lingual), using compressed air to dry the teeth, cotton rolls to remove debris where necessary, a dental mirror, probe and optimal light. The observations were dictated to a dental nurse who recorded the scores in a diagram. An individual mean VEDE-score was calculated by summing up the highest surface score for each tooth divided by the number of teeth present. This was used in the data analyses.

Before using the VEDE scoring system, calibration using 74 surfaces from intraoral close-up photographs in a training program [33] was performed by four examiners. Interobserver agreement was 0.68 expressed as linear weighted Cohen’s kappa (K_w_) [36]. To further test the reliability of the principal observer the same observers scored intraoral photos and dental casts using the VEDE-index [33] on non-occluding surfaces (of all study participants, from distal surface of upper right canine tooth to distal surface of upper left canine tooth) (Table 1). Interexaminer agreement (K_w_) in the evaluations of VEDE was 0.51. The scores by RS were close to the mean of the four examiners.

**Table 1:** Tooth wear presented as mean VEDE by four examiners

**Examiners**	**VEDE**^**a**^		
	**PWS**	**Control**	
I	1.50 (±1.5)	0.48 (±0.7)
II	0.57 (±1.0)	0.12 (±0.3)
III	1.14 (±1.1)	0.38 (±0.5)
RS	1.15 (±1.1)	0.18 (±0.3)

^a^ Mean (standard deviation).

Tooth wear on occluding surfaces was evaluated by four examiners on dental casts and intraoral photographs using the I_A_ index. This index recorded tooth wear on a 4-point scale: 0 = no or minimal wear; 1 = wear of enamel down to dentine spots; 2 = wear of the dentine down to one-third of the crown height; 3 = wear of the dentine greater than one-third of crown height. In this study, the presence of a dental prosthetic crown due to tooth wear (according to the dental records) also qualified for a score of 3. The individual tooth wear index (I_A_) was calculated using the following formula: (10 G_1_ + 30 G_2_ + 100 G_3_)/(G_0_ + G_1_ + G_2_ + G_3_), where G_0_, G_1_, G_2_ and G_3_ = number of teeth with occlusal wear scores of 0, 1, 2 and 3 respectively [34]. Before evaluating tooth wear, the examiners were calibrated and blinded examinations were performed. The mean score from the four examiners was used to calculate the severity of incisal and occlusal wear (I_A_). Interexaminer agreement (K_w_) in the evaluations of I_A_ was 0.67. The modified I_A_ was logarithmically transformed to render a more symmetrical distribution of data in regression analyses.

Unstimulated whole saliva (UWS) was collected and salivary volumes determined by weight (1 g = 1 ml). The flow rates are presented as ml/min. Results of the saliva analyses have been presented elsewhere [24]. However, as this information may be relevant for tooth wear, the UWS values were included in the present analyses. BMI calculated on the basis of measured height and weight, was age- and gender adjusted [37] and is presented elsewhere [24]. Three participants from the study group who had extensive tooth wear and cooperated well with the examination procedures, were referred to 24-h oesophageal pH-metry.

### Statistical analysis

The interexaminer agreement was measured using linear weighted Cohen’s kappa. Calculations were based on raw data. A two-sided *t*-test was used to compare VEDE between the two groups, whilst a two-sided Mann–Whitney test was used to compare I_A_. The association between two continuous variables: tooth wear and age, and tooth wear and saliva secretion was analysed by Spearman correlation coefficient (r). To study the relationship between tooth wear (VEDE and log I_A_) and several variables simultaneously, linear regression analysis was performed. The following variables were significantly associated with tooth wear and became candidates for multivariate analysis; PWS (yes/no), age, tooth grinding (yes/no), and saliva secretion. A significance level of 5% was used throughout. The statistical analysis was carried out using the statistical software program SPSS© (v. 18.0, SPSS Inc., Chicago, III., USA).

## Results

**Figure 1:**
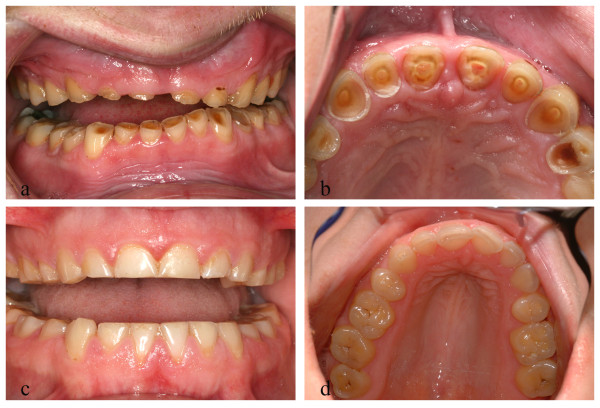
**Tooth wear in PWS.** (**a, b**) Extreme tooth wear with pulp exposure in 6 teeth in a 36-year-old male, (**c, d**) Typical tooth wear in a 28-year-old female.

Characteristics of the study population are presented in Table 2. Eighteen individuals with PWS and 3 controls reported tooth grinding. The number of individuals reporting high consumption of acidic food and drinks was much higher among individuals with PWS than in controls (Table 3). Twelve out of forty-nine individuals with PWS reported that they did not like and did not drink any water at all, whilst sixteen individuals drank some water as part of their fluid intake. Examples of extreme tooth wear are presented in Figure 1.

**Table 2:** Characteristics of the study population

	**PWS group (n = 49)**	**Control group (n = 49)**
Age range (years)	6.5-40.9	6.1-42.5
Mean age ± s.d.	20.4 ± 9.5	20.8 ± 10.1
Number of individuals	n	n
Age group 6-18 years	23	23
Age group 19-40 years	26	26
Males/females	25/24	25/24
**Supplementary diseases**		
Diabetes	3	0
Epilepsy	3	0
Heart disease	1	0
Daytime sleepiness or Sleep apnea	20	0
**BMI**		
Normal weight	14	49
Overweight	17	0
Obese	18	0
**Medication**		
Growth hormone (GH) ^a^	42	0

BMI criteria were age- and gender adjusted for individuals 6-18 years according to International Obesity Task Force (IONT) (37).

^a^Duration of GH treatment ranged between 6 and 192 months. Mean duration was 72 months.

**Table 3:** Number of individuals with high consumption^*^of selected acidic drinks and foods

	**PWS (n = 49)**	**Controls (n = 49)**	**p-value**
**Drinks**			
Sugared soft drinks carbonated/not carbonated	1	4	0.17 ns
Diet soft drinks carbonated/not carbonated	30	1	<0.001
Natural fruit juice	10	15	0.25 ns
**Foods**			
Fruit	36	27	0.06 ns
Yoghurt	16	7	0.03

^*^Combines the frequencies: ‘daily’ and ‘several times a day’.

**Figure 2:**
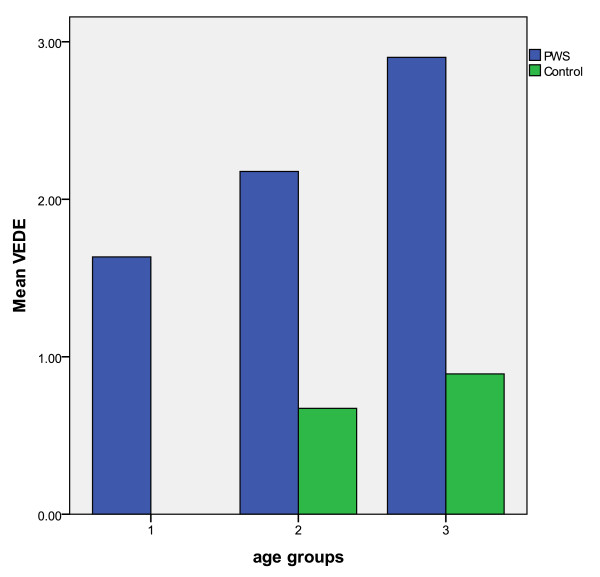
**Tooth wear presented as mean VEDE for individuals in PWS- and control-group divided in three equally large age groups.** Age groups (mean age, range years): 1; 9.8 (6.1-13.6) (n = 32), 2; 20.2 (13.7-25.4) (n = 34), 3; 31.5 (25.5-42.5) (n = 32).

According to the VEDE-scoring system (clinical examination by RS), five individuals with PWS and seven controls did not have tooth wear. Mean VEDE score was 1.70 ± 1.44 in the PWS group and 0.46 ± 0.36 in the control group (p < 0.001). Twenty-nine individuals with PWS and 13 controls presented with tooth wear exposing dentine (score 3, 4 and 5) or had dental crowns due to severe tooth wear. Tooth wear in three different age groups and according to mean VEDE-score is presented in Figure 2.

**Figure 3:**
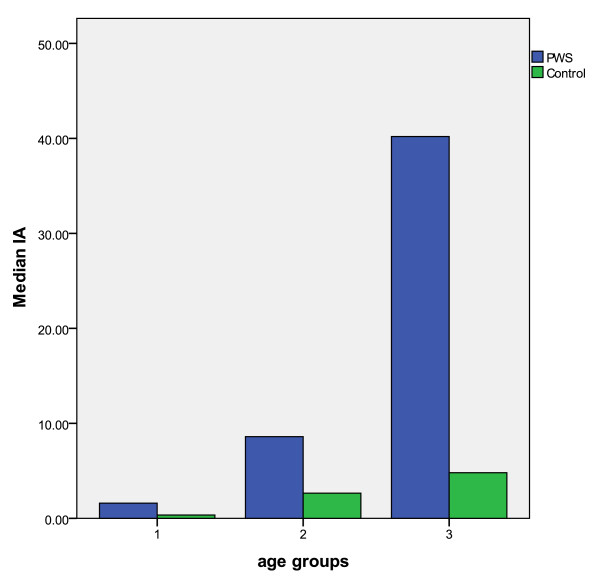
**Tooth wear presented as median I**_**A**_**for individuals in PWS- and control-group divided in three equally large age groups.** Age groups (mean age, range years): 1; 9.8 (6.1-13.6) (n = 32), 2; 20.2 (13.7-25.4) (n = 34), 3; 31.5 (25.5-42.5) (n = 32).

Four examiners evaluated tooth wear (presented as median I_A_) from dental casts and intraoral photographs (Table 4). No tooth wear according to the I_A_ index (I_A_ = 0) was recorded in four individuals with PWS and in six controls. Median I_A_ was 7.50 (2.60-30.70) in the PWS group and 2.60 (0.90-4.70) among controls (p < 0.001). Median I_A_ in three age groups is presented in Figure 3.

**Table 4:** **Tooth wear presented as median I**_**A**_**by four examiners**

**Examiners**	**I_A_^a^**		
	**PWS**	**Control**	
I	10.40 (3.8-48.0)	4.40 (1.3-6.9)
II	3.60 (0.2-18.3)	0.40 (0-2.0)
III	6.50 (2.4-28.0)	2.90 (0.7-5.5)
RS	7.50 (3.1-26.6)	2.10 (0.8-3.8)

^a^ Median (interquartile range).

Mean unstimulated whole salivary flow rate in the PWS group was 0.12 ± 0.11 ml/min as compared to 0.32 ± 0.20 ml/min in the control group (p < 0.001).

In the PWS group, tooth wear correlated significantly with age (VEDE; r = 0.79, p < 0.001, I_A_; r = 0.82, p < 0.001) and saliva secretion (VEDE; r = 0.46, p = 0.001, I_A_; r = 0.43, p = 0.002). Comparing grinders and non-grinders mean VEDE was 2.67 ± 1.62 and 1.14 ± 0.97, p = 0.001 respectively. Corresponding median I_A_ values were 25.70 (5.48-68.55) vs. 5.70 (1.60-9.10) (p = 0.003). No significant association was found between tooth wear and obesity, gender or self-reported consumption of acidic drinks in the PWS group. The only factor associated with tooth wear in the control group was age (VEDE; r = 0.77, p < 0.001, I_A_; r = 0.71, p < 0.001). The association between tooth wear and obesity or tooth grinding could not be evaluated in the control group, because there were no obese individuals and only 3 individuals reported tooth grinding. Multivariate linear regression analysis was performed with tooth wear as the dependent variable and PWS (yes/no), age, tooth grinding and saliva secretion as independent variables. PWS (yes/no), age and tooth grinding retained a significant association with VEDE (p < 0.001) and log I_A_ (p < 0.001). The difference in mean VEDE between the PWS- and control group was 1.25 (CI 0.83-1.67) without adjustment, and 0.88 (CI 0.49-1.26) when adjusting for age, tooth grinding and saliva secretion. Similarly, the difference in mean log I_A_ between the PWS- and control group was 1.04 (CI 0.60-1.48) without adjustment and 0.69 (CI 0.32-1.06) when adjusting for age, tooth grinding and saliva secretion. The mean difference in VEDE score and median difference in I_A_ score between the PWS and controls remained significant (p < 0.001) when tooth grinders were excluded.

Clinical examination showed that six individuals in the PWS group had one or more dental crowns and three of them had all their teeth crowned due to tooth wear (according to previous dental records). The examination triggered referrals for prosthodontic treatment for 4 participants (M/23, F/24, M/28, M/36). Due to extreme tooth wear (some with pulp involvement), all teeth in these individuals were treated with dental crowns. No study participants or controls reported oral acid/burning sensation or a medical history of gastro-oesophageal reflux disease (GORD). Twenty-four-hour oesophageal pH-metry revealed severe pathological reflux in all three individuals in the study group (n = 2 > 18 years, n = 1 < 18 years).

## Discussion

To our knowledge this study is the first to evaluate tooth wear in Prader-Willi syndrome and compare it with a control group. Results from our study demonstrate extensive tooth wear in PWS compared with the control group, and the null hypothesis was therefore rejected. Tooth grinding was significantly associated with tooth wear and could explain it to some extent.

Tooth wear may be caused by either attrition, erosion, abrasion or a combination of these conditions. Reviewing the literature on tooth wear can be confusing as there seems to be lack of standardization of indices used in epidemiological studies on dental wear. No index seems to be ideal for use in epidemiological prevalence studies [38]. In the present study two indices have been used to focus on erosive wear (VEDE) as well as abrasion/attrition on occluding surfaces (I_A_). A large number of indices have been developed for diagnosing, grading and monitoring tooth wear. Some researchers have recorded lesions on an etiological basis (erosion indices) [39-42], whilst others have recorded lesions irrespective of etiology (tooth wear indices) [38,43]. Knowledge regarding the validity of current diagnostic criteria for different forms of tooth wear is incomplete and further research is needed [44]. The prevalence of tooth wear in healthy children and adolescents has been investigated using erosion indices in several studies [40,45,46]. Attrition is often considered to be the cause of wear of incisal and occlusal surfaces, although erosion may also be an important factor. The individual tooth wear index (I_A_) used in our study scores wear of both natural teeth and restorative materials, stating the ratio between the weighted sum of all teeth with some degree of tooth wear and the total number of teeth in that individual. The tooth wear may have been overestimated in individuals with PWS in this study as all teeth covered with dental crowns due to tooth wear were given a score of 3 (highest possible attrition score). Some of these teeth may have scored only 2 before treatment.

All study participants and controls were examined once by a single examiner (RS). Repeated clinical examinations (using two or more examiners) were not possible due to the significant travel distance to the clinic for many of participants. To counter this potential bias, 4 calibrated and blinded examiners evaluated tooth wear based on the VEDE- and I_A_ indices using dental casts and intraoral photographs. The VEDE index is based on 6 categories whilst the I_A_ index involves 4. The low level of interexaminer agreement demonstrates reliability issues in the use of these scoring indices for erosive wear. This is in accordance with reports from other studies [47,48]. Due to the multifactorial causes of dental wear it is difficult for one examiner to establish the etiology in each case. Erosion and wear are related, but in occluding surfaces the wear component might be considered as the main factor while erosion could be the most prominent cause of substance loss in non-occluding free surfaces. The two indices chosen, facilitates the monitoring of these aspects. The results showed consistently that individuals with PWS were more affected than the controls. In addition the principal examiner RS, was an average observer which indicate acceptable validity of the clinical recordings.

Moderate tooth wear may progress throughout life as part of normal aging. Few data are published describing levels of tooth wear according to age. Severe tooth wear has been reported to increase from 3% at the age of 20 to 17% at the age of 70 [49]. The occurrence of age-related physiological tooth wear is controlled for in the present study as the PWS and control group are age-matched.

Within the present study design the associations identified are not necessarily indicative of causality. However, several clinical findings may be of significance. Hyposalivation was found in 54% of individuals in the PWS group and 3% in the control group [24]. Saliva protects the teeth against tooth wear [32,50] as a result of its ability to form the acquired enamel pellicle which acts as a lubricant to reduce frictional wear. Also its buffering capacity, its clearance and dilution of acids in the mouth protect against enamel dissolution. In the PWS group tooth wear was associated with salivary flow rate. However, salivary flow rate did not maintain a significant association with tooth wear in the multivariate regression analysis. Hyposalivation could therefore be a minor factor contributing to the extreme tooth wear seen in individuals with PWS.

Unusual water intake and drinking behavior have been observed in people with PWS. Such behavior may reflect a hypothalamic alteration [51]. Most of the investigated study participants reported disliking water in infancy. Individuals with PWS, their parents and carers appeared to be well-informed on this issue, and described conscious efforts to teach their children to drink water from an early age.

Dental erosion is caused by the presence of intrinsic (gastro-oesophageal reflux) and/or extrinsic (most commonly dietary) acid of non-bacterial origin in the mouth [52]. Measurement of consumption of acidic food and drinks was based on self-report during the anamnestic interview, and information bias may therefore be a limitation in the present study. Data analysis revealed no statistically significant association between erosive wear and high consumption of acidic food and drinks. These results are in accordance with a study on a healthy population in Iceland [40]. Nevertheless, the role of dietary acids is considered to be the most common cause of erosive wear by many researchers [41,42,53,54]. The primary risk factor with dietary acids is high frequency of consumption. The dietary acids cause an immediate drop in oral pH, which returns to physiological pH within a couple of minutes due to the neutralizing effect of saliva. Eating behaviors associated with PWS suggest that these individuals suffer from persistent hunger and a consequent urge to eat [55]. When they are given access to soft drinks it is therefore unlikely that drinking of the beverage is spread over time. This may go some way towards explaining the lack of association between high consumption of acid drinks and erosive wear.

In our study tooth grinding was recorded according to self-report or report by parents/carers. Given that the activity is thought to be cyclic and a parent or carer may not be in sufficiently close proximity to detect audible tooth grinding, some bruxing may be undetected, leading to underestimation of its true level. The frequency and intensity over time are seldom known. Reported tooth grinding varied significantly between the study group and control group. However, tooth grinding is unlikely to be the only cause of tooth wear as the significant difference between the two groups remained when tooth grinders were removed from the analysis. Similar findings have been reported by others [34,56]. The significance of tooth grinding as a causative factor is not fully known, but has probably been overestimated previously [57]. The multifactorial etiology of tooth grinding is well-recognized. Potential contributors include psychological factors, sensitivity to stress, and arousals during sleep, with accompanying increases in heart rate, muscle activity, and physical movements [58,59]. The abnormal and frequent sleep arousals [60] and characteristic behaviors including self-harm and sensitivity to stress documented in PWS [61] may lead to tooth grinding as a sequela of the syndrome.

Gastro-oesophageal reflux (GORD) is an aspect of general health which may affect erosive wear. GORD is a significant intrinsic factor in dental erosion [42,62-64]. Studies have shown a significant increase in reflux symptoms in individuals with confirmed obstructive sleep apnea syndrome and increased BMI [65,66]. Central adiposity may be the most important risk factor for development of reflux [67]. In our study, 10 of 13 patients referred to polysomnography were diagnosed with obstructive sleep apnea syndrome and 37% were obese with central obesity, both defined as risk factors in association with GORD. No study participants reported symptoms of reflux or heartburn, although all three examined individuals had severe reflux requiring medication. Symptoms of gastric reflux may be underreported because high pain threshold in PWS may lead to decreased recognition of injury or illness [7,68,69]. Alternatively individuals with the disorder may regard their long-standing reflux symptoms as “normal”. The prevalence of GORD associated with extreme tooth wear in the three patients referred for reflux assessment suggests that GORD may be an etiological factor worthy of further investigation.

The need for prosthodontic rehabilitation is high in young adults with PWS. Three study participants (M/24, M/27, M/40) had dental crowns on all their teeth at examination as a result of extreme tooth wear. Following the examination, four others (M/23, F/24, M/28, M/36) required referral to a prosthodontist to have all teeth treated with dental crowns. This means that seven of 26 adults in the study group (27%) had all their teeth crowned between the age of 22 and 36 years. One adult study participant needed endodontic treatment prior to prosthodontic treatment due to pulp exposure in 6 teeth. He had never complained of pain or sensitivity to cold or heat. High pain threshold in individuals with PWS is recognized [68,69] and should be taken into account in the examination and treatment of individuals with PWS. Tooth wear is a multifactorial injury and the cause may vary between individuals. Despite the limitations of the present study due to its study design, the results highlight the strength of association between possible causative factors and tooth wear in PWS. Preventive treatment is important in this group. Dental splints, for example, may be useful for some, although they may be poorly-tolerated by others. Reducing dietary consumption of acid and replacing soft drinks with water may also protect against tooth wear. Tooth wear should be closely monitored by the dental team to facilitate early diagnosis. Regular follow up is necessary to assess the need for timely restorative treatment.

## Conclusion

Our study provides evidence that tooth wear, in terms of both erosive wear and attrition, is a significant problem in Prader-Willi syndrome and young adults have a considerable need for prosthodontic rehabilitation.

## Competing interests

The others declare that they have no competing interests.

## Authors’ contributions

RS developed the study design and the study protocol in cooperation with IE, KS, LS and HN. She established the primary aims in consultation with the coauthors. RS has been responsible for all data collection and analyses and for ensuring that the results are interpreted and presented correctly. She carried out literature searches and identified relevant literature. RS has written the paper in consultation with the coauthors and takes full responsibility for the content. IE contributed to the conception and design of the study. He assisted with database management and contributed to the analyses of the data. He critically reviewed the paper and approved the final version. KS as the primary supervisor, contributed to the development of the overall study design, its aims and implementation. She critically reviewed the paper and approved the final version. LS assisted in the development of the study design. He supported RS with the statistical analyses and interpretation of the data. He was actively involved in writing the statistical analyses section of the paper. He critically reviewed the paper and approved the final version. HN contributed to the development of the study design and the aims of this paper. She also contributed to the analyses and to the interpretation of saliva data. She critically reviewed the paper and approved the final version.

## Authors’ information

RS, HN and KS work at the National Resource Centre where patients with rare conditions (< 1:10000) are referred from the whole country. With the support of our specialists we collect, systematize and disseminate knowledge about oral health problems in rare conditions. We examine patients, suggest treatment plans, teach students and give courses. Research is an important part of our activities.

## Pre-publication history

The pre-publication history for this paper can be accessed here:

http://www.biomedcentral.com/1472-6831/12/12/prepub
